# Giant right ventricular myxoma presenting as right heart failure with systemic congestion: a rare case report

**DOI:** 10.1186/s12893-020-00977-4

**Published:** 2021-01-29

**Authors:** Chen Lu, Peng Yang, Jia Hu

**Affiliations:** Department of Cardiovascular Surgery, West China Hospital, Sichuan University, No.37 Guoxue Alley, Wuhou District, Chengdu, 610041 Sichuan Province People’s Republic of China

**Keywords:** Right ventricular myxoma, Right heart failure, Systemic congestion, Surgery

## Abstract

**Background:**

Myxoma is an uncommon disease and its symptoms vary greatly depending on size, location and mobility. Right-sided myxoma, especially right ventricular myxoma, is much rarer, and the symptoms are alien and uncharacteristic. The lack of understandings poses challenges to prompt diagnosis and timely treatment.

**Case presentation:**

A 44-year-old female patient was diagnosed with giant right ventricular tumor. Right heart failure and systemic congestion caused by right ventricular outflow tract obstruction were observed on this case. Surgery was performed to excise the mass which was measured at 9.5 * 5.0 cm and confirmed as myxoma pathologically.

**Conclusions:**

Right-side myxoma is easy to be unnoticed due to its low incident rate and atypical symptoms. Delay in surgical intervention might cause unrecoverable complications. More comprehensive understanding of the symptoms is expected to help improving the diagnose and treat of right-sided myxoma.

## Background

Primary tumors of the heart, mostly benign, are uncommon with an overall incidence of 0.0017 to 0.19% [1], and the majority of these benign neoplasms are myxomas localized in the left (75–85%) or right (15–20%) atrium [1,2]. Only rare cases have been reported of myxomas originating from ventricular chambers (2.5–4%) [1–3]. Moreover, the clinical symptoms of cardiac myxomas vary greatly on a case-to-case basis [2–5], largely depending on their size, location and mobility. A peripheral embolic event in patients without any conventional risk factors may prompt a suspicion of a left-sided cardiac myxoma [6]; however, the manifestations of a right-sided cardiac myxoma are often uncharacteristic, leading to delayed diagnosis, treatment and unfavorable outcomes [4,5]. Herein we report a very rare case of a giant right ventricular myxoma presented with right heart failure with systemic congestion. In addition, a comprehensive understanding of epidemiology, clinical characteristics of a right-sided cardiac myxoma is extremely important for prompt diagnosis and timely treatment.

## Case presentation

**Fig. 1:**
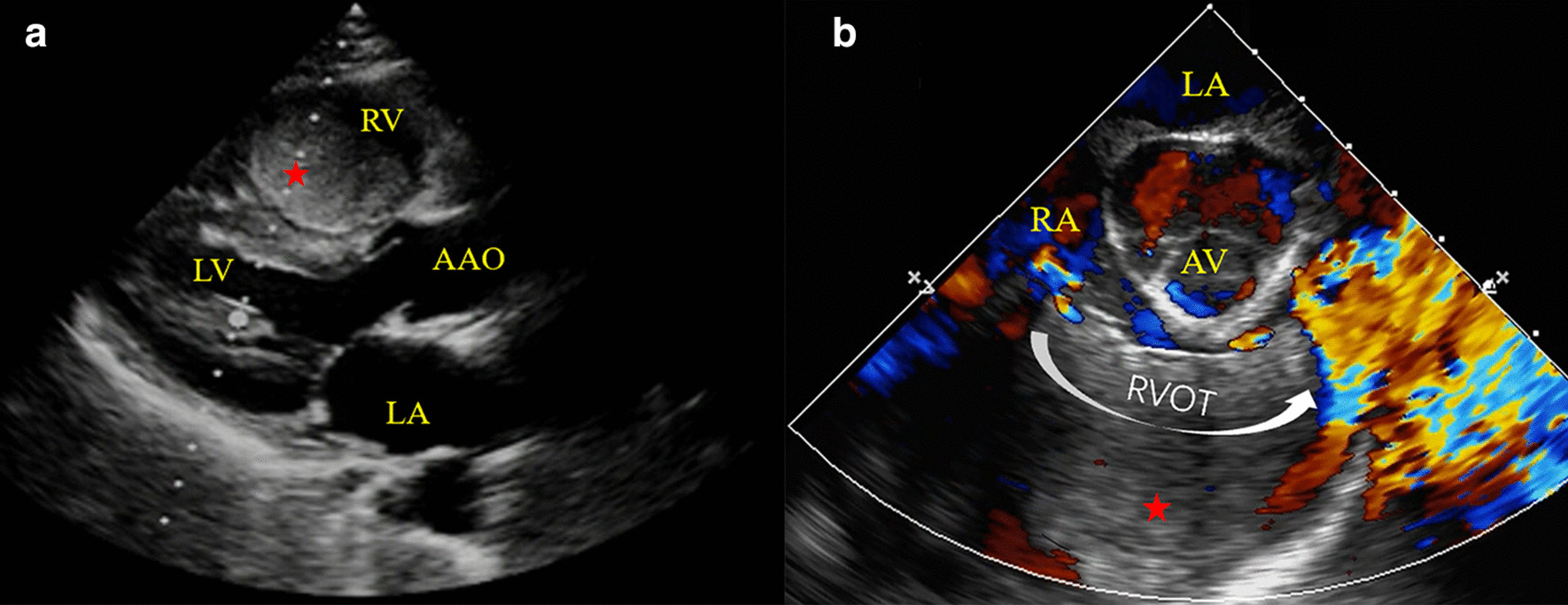
**a** transthoracic echocardiography before surgery showed a giant tumor (red star) occupied the majority of RV; **b** Transesophageal echocardiography (ME RV inflow-outflow tract) showed the tumor (red star) in right ventricular caused outflow obstruction. *LA* left atria, *LV* left ventricle, *RA* right atria, *RV* right ventricle, *AAO* ascending aorta, *AV* aortic valve, *RVOT* right ventricular outflow tract

**Fig. 2:**
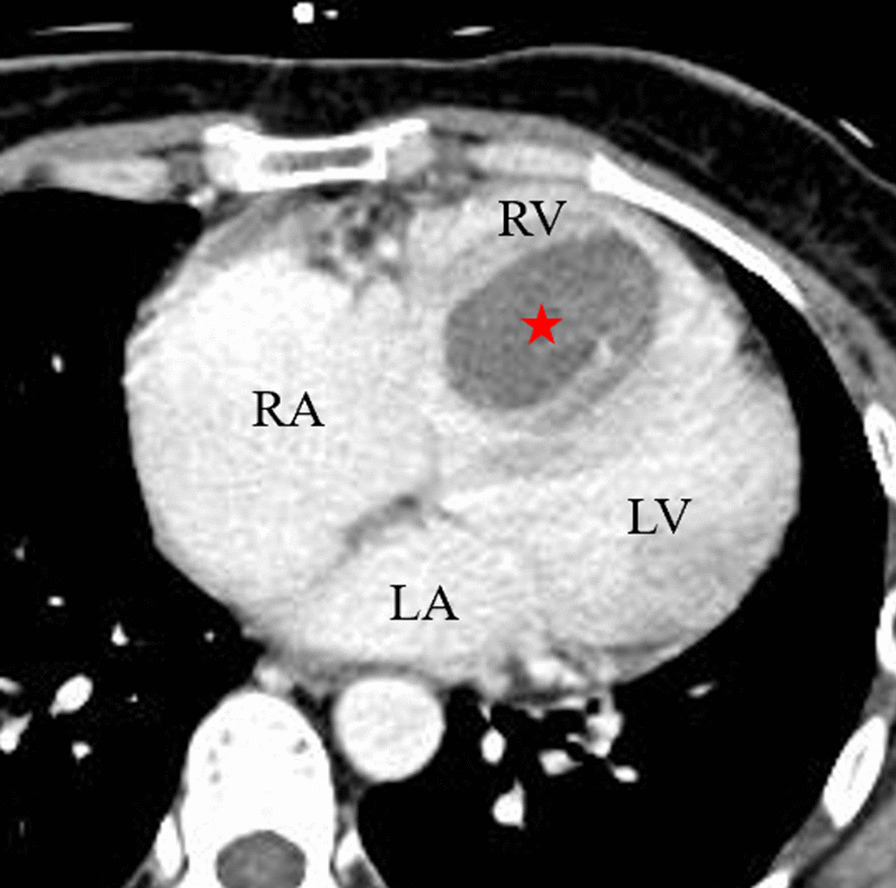
Contrast-enhanced computed tomography showed the tumor in RV had no contrast effect (red star); *LA* left atria, *LV* left ventricle, *RA* right atria, *RV* right ventricle

A 44-year-old female was admitted due to abdominal distension and persistent lower limbs edema for over 2 months. Other right-heart-failure-like symptoms caused by systemic congestion were also observed. Transthoracic echocardiogram (TTE) and transesophageal echocardiogram (TEE) found a giant phyllodes mass completely occupies right ventricle (RV, Fig. 1a) and causes right ventricular outflow tract (RVOT) obstruction (Fig. 1b). Contrast-enhanced computed tomography showed an irregular and slightly hypodense shadow (6.2 cm × 3.9 cm) with clear border (Fig. 2) in RV. MRI calculated ejection fraction of RV has sharply decreased compared with that of the left ventricle (24.1% vs. 55.3%) (Additional File 1).

**Fig. 3:**
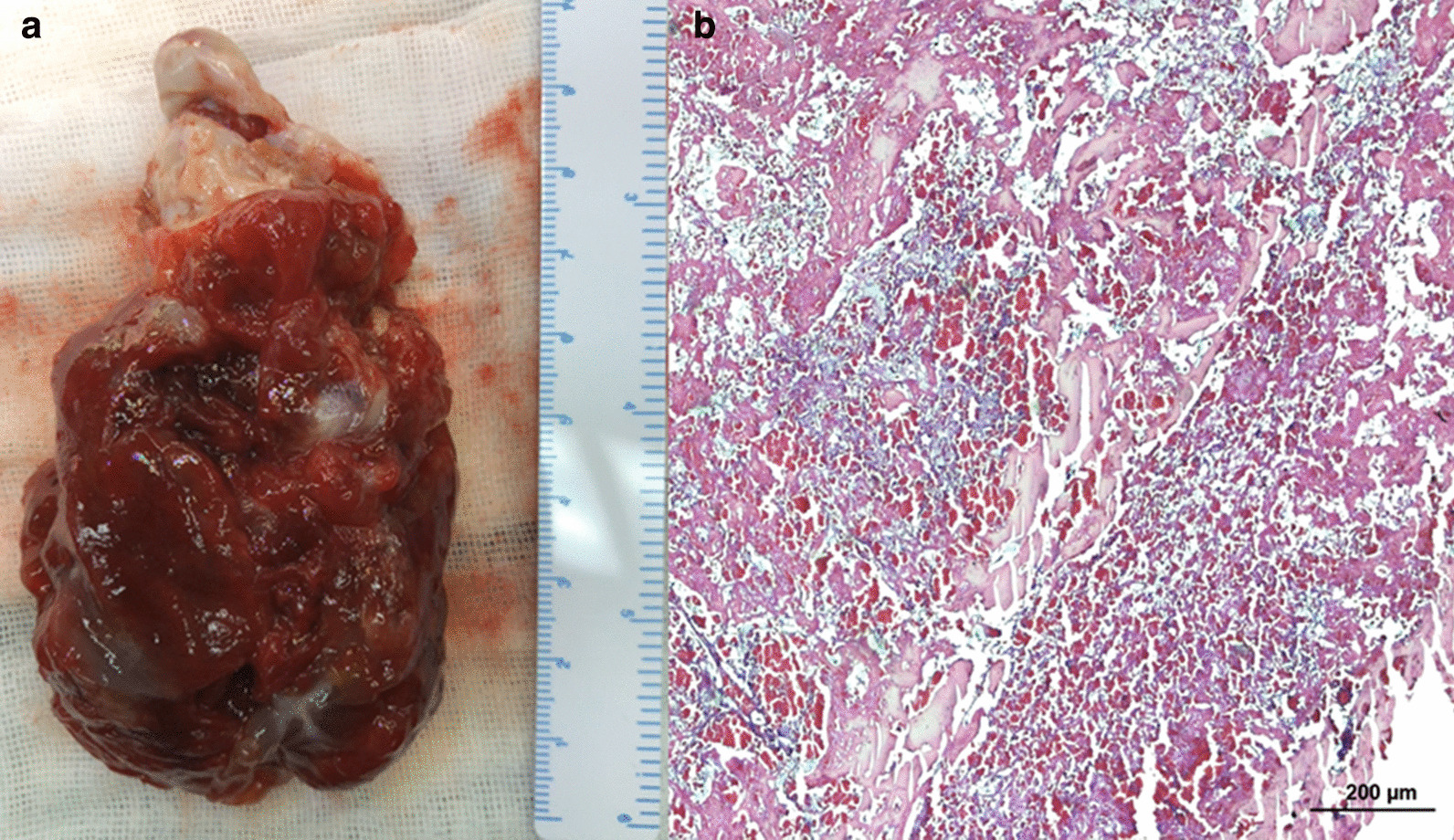
**a** macroscopic appearance of right ventricualr tumor (9.5 cm * 5.0 cm) showed a multinodular, pedicled, kermesinus mass with clear border; **b** the mass consisted of areas of stellate cells surrounded by abundant, loose, myxoid stroma microscopically

**Fig. 4:**
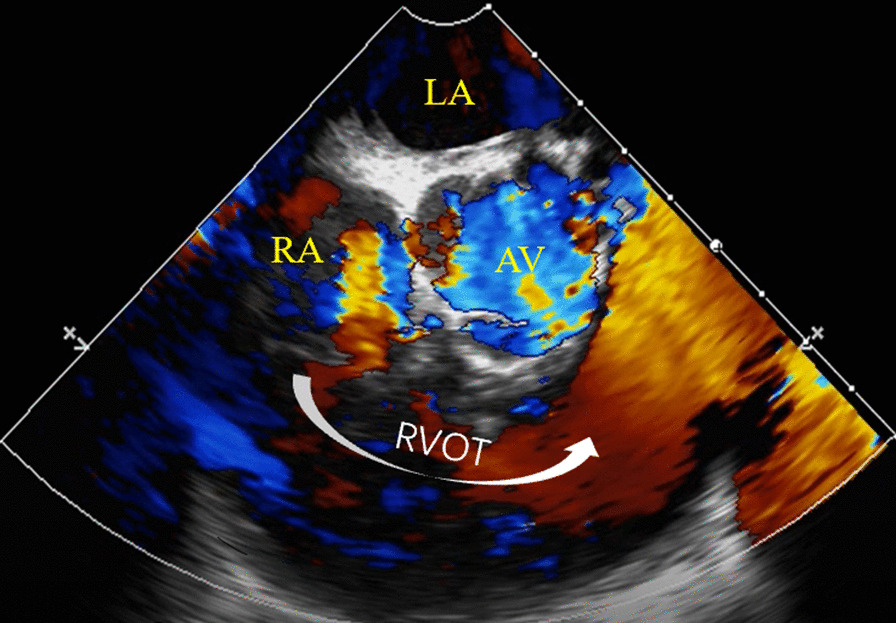
Transesophageal echocardiography (ME RV inflow-outflow tract) after surgery showed the tumor and outflow obstruction disappeared. *LA* left atria, *RA* right atria, *AV* aortic valve, *RVOT* right ventricular outflow tract

The patient underwent a surgery through a median sternotomy with cardiopulmonary bypass and a longitudinal right atriotomy was used to open the heart. A wide base mass originated from the right ventricle free wall and occupies most of the right ventricular cavity. No adhesion to the septal leaflet or chordae tendineae of the tricuspid valve. The tumor was completely excised from its base. The fundus was then cauterized and rinsed. The 9.5 * 5.0 cm neoplasm was a soft, pedicled and well-defined kermesinus mass (Fig. 3a). Microscopically, stellate cells can be seen scattered in a loose myxoid stroma (Fig. 3b). The diagnosis of right ventricular cardiac myxoma was hence confirmed. The patient recovered uneventfully and the pre-discharge echocardiogram showed no mass (Fig. 4). The patient was followed up for one year, no recurrence was found and the general condition was good.

## Discussion and conclusions

The occurrence of primary cardiac tumors is relatively low with more than 50% of which are myxomas [2]. Epidemiology data shows that the right-sided myxoma is less common than the left-sided ones [1,2]. Meanwhile, ventricular myxoma, which has a gross morbidity of less than 0.002% in the general population [7], is more uncommon than atrial myxoma [1–3]. Reported here is a giant right ventricular myxoma case which is extremely rare and very unlikely to be early catched due to its asymptomatic nature in its early stages (Table 1).

**Table 1:** A comparison of right/left sided myxoma

	Right-sided myxoma	Left-sided myxoma
Morbidity [1–3,7]		
Atrium	15–20%	75–85%
Ventricle	2.5–4%	3–4%
Symptoms		
Obstructive	Right heart failure: systemic congestion [4]: peripheral edema, ascites, superior vena cava syndrome	Left heart failure: pulmonary congestion: dyspnea [9] Systemic circulation ischemia: syncope [10]
Embolic	Pulmonary embolism [13]	Peripheral embolism [11,12] Cerebral/mesenteric/limb ischemia
Constitutional	Fever, Infected cardiac myxoma, weight loss, etc. [2]	
Onset [14]	Late onset	Early onset
Treatment		
Surgical resection	Timely surgical resection is always necessary for all myxomas [15,16]	
Prognosis		
No surgery	More than 8% of patient died of myxoma associated complications [17]	
Surgery	Operative mortality is less than 5% [15]	
Recurrence	Long-term recurrence is rare [18]	

The clinical manifestations of ventricular myxoma can be categorized into a classic triad including obstructive, embolic and constitutional [2]. Constitutional symptoms include fever, weight loss, infections and so on, which may be attributed to the release of cytokine interleukin-6 (IL-6). Obstructive symptoms are usually associated with the resistance that any chamber or valve orifice have against blood flow [8]. Obstructive and embolic symptoms are totally different between left-sided and right-sided cardiac myxoma. For patients with left-sided myxomas, obstruction may lead to pulmonary congestion or systemic circulation ischemia, in which case dyspnea [9] or syncope [10] may be the chief complaint. Whereas for patients with right-sided myxomas, peripheral edema, ascites, or superior vena cava syndrome which supposed to be caused by systemic congestion, are more common [4]. Embolic symptoms would be determined by the site of the embolization, which are essentially decided by the position of the myxomas. Left-sided myxoma may cause peripheral embolism [11,12] such as stroke, while right-sided myxoma can potentially cause pulmonary embolism [13].

The myxoma as reported in this case is very rare in terms of both location and size. It locates in the right ventricle only and is unprecedently large, to the best of our knowledge. Compared with myxomas in the left, manifestations of right-sided myxoma are more atypical and more difficult to be identified in an early stage, as its clinical signs can unveil six years later than did the left-sided ones [14]. The belated diagnosis due to late-onset appearance may result in pernicious outcomes. The only manifestation of the patient in this case is unperceived edema in the lower limbs. Even though the symptom had appeared for only two months, the huge myxoma had already caused severe RVOT obstruction and right heart failure with systemic congestion. In such a case, RVOT obstruction rather than inherent pump failure should be responsible for the right-heart-failure-like symptoms [4]. Once the myxoma was surgically resected in time, those symptoms would disappear without severe consequences.

Once a cardiac neoplasm was discovered, excision through open heart surgery is always recommended [15] for the following reasons. First, pathological diagnosis is crucial in making differentiation between benign and malignant lesions, as imaging exam alone cannot rule out the malignancy cases. Second, cardiac neoplasms can cause obstructive, embolic and constitutional symptoms, which may increase the patient’s risk of sudden death, stroke, organ infarction and other severe complications. Last but not least, small benign tumors may have no obvious symptoms but can grow rapidly and silently. The growth rate of myxoma is rarely monitored, a previous study [16] had reported a myxoma grew at a rate of 3 mm per month. Even for smaller neoplasm, benign or malignant, early resection could avoid severe complications in the long run.

Surgical resection is the only meaningful way to improve the long-term prognosis of cardiac myxoma. Even when awaiting to accept surgery, more than 8% of patients died of obstructive, embolic or other complications [17]. The surgical mortality is reported to be less than 5%, meanwhile the rate of ventricular myxomas is slightly higher than that of atrial myxomas [15]. Recurrences usually occur in the first 4 years after surgical excision and the overall recurrent rate is rare [18], which emphasizes the importance of fellow-up echocardiogram.

In summary, the timely diagnosis of right-sided myxomas is challenging due to the disease’s low incidence rate and nature of various, atypical symptoms. Hence, the treatments are tended to be delayed and the prognosis are not so good as a result. The recommendation that merely diagnosis and surgical resection in time could reduce the risk of malignant potential, embolism and obstruction makes an emphasis on the necessity of thorough understanding of right-sided cardiac myxomas. As timely diagnosis followed by proper surgical intervention could significantly decrease the chances of severe outcomes in patients with right sided cardiac myxomas, further studies focusing on the early diagnosis and treatment techniques of right-sided cardiac myxomas could be very meaningful.

## Supplementary information

**Additional File 1:** This additional file is a video containing the patient’s preoperative and postoperative CT, MRI and echocardiography.

## Data Availability

The datasets supporting the conclusions of this article are included within the article.

## Abbreviations

AAO: Ascending aorta
AV: Aortic valve
LA: Left atria
LV: Left ventricle
RA: Right atria
RV: Right ventricle
RVOT: Right ventricular outflow tract
TEE: Transesophageal echocardiogram
TTE: Transthoracic echocardiogram
