# Supplementary figures and images for: Giant right ventricular myxoma presenting as right heart failure with systemic congestion: a rare case report
Source: BMC Surg. 2021 Jan 29;21:64. doi: 10.1186/s12893-020-00977-4 (PMC7845124; doi:10.1186/s12893-020-00977-4)

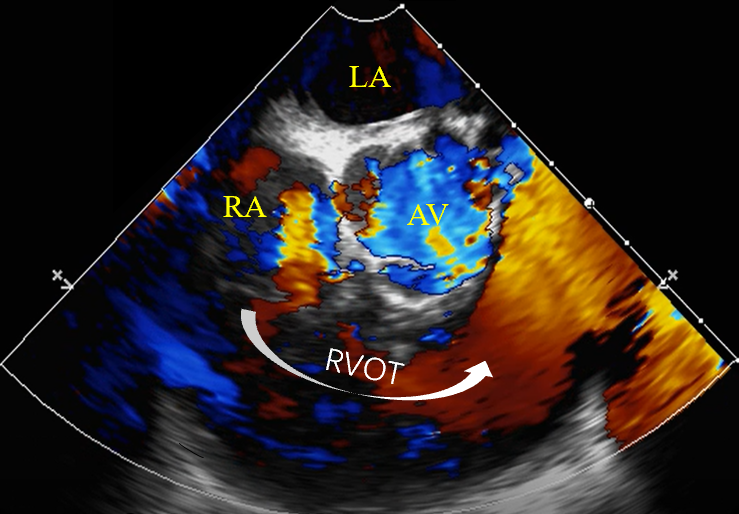

Supplement: Supplementary file 1 — Additional File 1: This additional file is a video containing the patient’s preoperative and postoperative CT, MRI and echocardiography. [file 12893_2020_977_MOESM1_ESM.bmp]
